# Alcohol dependence and treatment utilization in Europe – a representative cross-sectional study in primary care

**DOI:** 10.1186/s12875-015-0308-8

**Published:** 2015-07-29

**Authors:** Jürgen Rehm, Allaman Allamani, Zsuzsanna Elekes, Andrzej Jakubczyk, Jakob Manthey, Charlotte Probst, Pierluigi Struzzo, Roberto Della Vedova, Antoni Gual, Marcin Wojnar

**Affiliations:** Centre for Addiction and Mental Health, 33 Russell Street, Toronto, ON M5S 2S1 Canada; Addiction Policy, Dalla Lana School of Public Health, University of Toronto, 155 College Street, 6th floor, Toronto, ON M5T 3M7 Canada; Institute of Medical Science, University of Toronto, Faculty of Medicine, Medical Sciences Building, 1 King’s College Circle, Room 2374, Toronto, ON M5S 1A8 Canada; Department of Psychiatry, University of Toronto, 250 College Street, 8th floor, Toronto, ON M5T 1R8 Canada; Institute of Clinical Psychology and Psychotherapy & Center of Clinical Epidemiology and Longitudinal Studies (CELOS), Technische Universität Dresden, Chemnitzer Str. 46, 01187 Dresden, Germany; Agenzia Regionale di Sanità Toscana, Villa la Quiete alle Montalve, Via Pietro Dazzi 1, 50141 Firenze, Italy; Corvinus University of Budapest, Közraktár u. 4-6, H-1093 Budapest, Hungary; Department of Psychiatry, Medical University of Warsaw, Nowowiejska 27, 00-665 Warsaw, Poland; Regional Centre for the Training in Primary Care (Ceformed), Via Galvani 1, 34074 Monfalcone (GO), Italy; Department of Life Sciences, University of Trieste, Via Weiss, 2, 34128 Trieste, Italy; Center for Study and Research in General Practice (CSeRMEG), Via Praga, 22, 20052 Monza (MI), Italy; Addictions Unit, Psychiatry Department, Neurosciences Institute, Hospital Clinic, Carrer Villarroel 170, 08036 Barcelona, Catalonia Spain; Institut d’Investigacions Biomèdiques August Pi i Sunyer (IDIBAPS), Carrer Rosselló 149, 08036 Barcelona, Catalonia Spain; Red de Trastornos Adictivos (RTA - RETICS), Instituto de Salud Carlos III, Villarroel 170, 08036 Barcelona, Catalonia Spain; Department of Psychiatry, University of Michigan, 4250 Plymouth Rd, Ann Arbor, MI 48109 USA

**Keywords:** Alcohol dependence, Composite International Diagnostic Interview, General practitioner, Primary care, treatment, Co-morbidity, Liver disease, Disability, Mental distress

## Abstract

**Background:**

Alcohol dependence (AD) in Europe is prevalent and causes considerable health burden. Recognition by general practitioners (GPs) and provision of or referral to treatment may contribute to reduce this burden. This paper studied AD prevalence in varying European primary care settings and examined who received treatment.

**Methods:**

In a cross-sectional multi-centre study in six European countries, 358 general practitioners assessed 13,003 primary care patients between January 2013 and January 2014, of which 8,476 patients were interviewed, collecting information on socio-demographics, physical and mental problems, and on alcohol use, problems and treatment. AD diagnoses were determined by GPs’ clinical judgement and a standardized interview. A wide definition for AD treatment included individual and group interventions provided by different health professionals. Descriptive as well as inferential statistics were employed.

**Results:**

AD was prevalent among patients in European primary health care settings (8.7 %, 95 % confidence interval (CI): 8.1-9.3 %). Treatment rates were low (22.3 % of all AD cases, 95 % CI: 19.4-25.2 %). For both prevalence and treatment utilization, considerable country variations were observed. AD was associated with a number of socio-economic disadvantages (e.g. higher unemployment rate) and higher physical (e.g., liver disease, hypertension) and mental comorbidities (e.g., depression, anxiety). Liver problems, mental distress and daily amount of alcohol used were higher among treated versus untreated male patients with AD.

**Conclusion:**

A minority of people identified as having AD received treatment, showing heavier drinking patterns and a higher level of co-morbidity. Different types of treatment, depending on severity of AD, should be considered.

**Electronic supplementary material:**

The online version of this article (doi:10.1186/s12875-015-0308-8) contains supplementary material, which is available to authorized users.

## Background

### Rationale

Mortality and disease burden in Europe are considerably impacted by alcohol use disorders and in particular alcohol dependence (AD) [1, 2]. The Diagnostic and Statistical Manual of Mental Disorders (4^th^ Edition, DSM-IV) defines AD as a mental disorder [3] with marked clinically relevant impairments and functionality constraints. Unlike other mental disorders, AD has not only shown to be linked to a high level of disability [4], but also to a high level of mortality, even in young adulthood [5, 6]. Despite the high level of mortality and disease burden associated, the treatment rate for AD in the adult population has been persistently low in Europe [1, 7–9]. The reasons for the low treatment rate are variable and understudied, and can be categorized into aspects related to the patient (e.g. attitudes, knowledge), the treatment system (e.g., availability, affordability, provider skills and knowledge), and to the larger environment [10, 11].

In order to increase treatment rates, primary care physicians or general practitioners (GPs) are considered as pivotal [12]. However, results of an international study including 7 European countries conducted by the World Health Organization in primary care centres suggests that AD recognition by GPs was low [12]. Severity of the disorder and associated disability have been shown to be positively associated with better recognition [12–14]. Even if identified, most patients with AD seem to receive no professional interventions [7], partly because the patients do not want treatment for their conditions, and partly because of low referral rates and/or because the GPs do not feel competent to initiate treatment themselves [15, 16].

### Research questions and objectives

The broad objective was to examine the level and nature of alcohol problems in general and AD in particular in primary health care facilities in six European countries. More specific objectives were threcognition of alcohol problems by primary health care physicians, and aspects related to interventions. In particular, the following research questions were underlying this study:

- What is the 12-month prevalence of AD in primary health care (by region and across regions), and how do people with an AD diagnosis differ from those without?
- What is the proportion of people with AD in treatment or receiving other interventions for their alcohol problems?
- What characteristics of the patient are linked to treatment provision?

## Methods

### Regions

The following regions and countries were part of the study, representing about 6.9 % of the EU population as whole [17]: Friuli-Venezia Giulia region (Italy1), Tuscany region (Italy2), Saxony and Berlin state (Germany), Hungary, Latvia, Łódzkie and Podkarpackie provinces (Poland), and Catalonia autonomous community (Spain). Countries were selected to include each of the three prototypical drinking pattern traditions in Europe [7, 18, 19], i.e., Mediterranean wine drinking cultures (Italy, Spain), middle European beer drinking cultures (Germany), and central and eastern-European countries with irregular heavy drinking occasions (Hungary, Latvia, Poland). In a second step, we drew nationally (smaller countries: Latvia, Hungary) and regionally representative (larger countries: Germany, Italy, Poland, and Spain) samples of primary care practices in these countries.

### Setting and participant sampling

Even though their exact role varies by country, primary health care practices are key to health care access in Europe. Typically, patients consult GPs for most of their health problems and receive basic interventions including, but not limited to prescriptions, and may be referred to specialists if needed. Sampling patients in the primary care practices was done between January 2013 and January 2014 on a predetermined day or consecutive days. The GPs were instructed to assess all patients aged 18 to 64 coming to their practice for a consultation in all countries. On average, GPs assessed 15.5 (95 % confidence interval (CI): 15.3-15.7) patients on a single day. Considerable mean differences between countries (minimum: 6.4 patients in Poland; maximum: 34.4 patients in Spain) and GPs (minimum: 1 patient per day; maximum: 53 patients per day) were present.

In Hungary and Spain, all patients consenting to study participation were assessed by their GP, and interviewed after their consultation. In Germany, all patients leaving their contact details with the GP after their consultation were asked for a subsequent interview. In the remaining countries (Italy, Latvia, and Poland), the GP assessment was used to determine subsamples to be interviewed. Here, risky drinkers were oversampled in order not to miss patients with AD. This procedure was determined a priori and sampling design was considered in all respective analyses.

### Variables and measurement

Details and flow of the GP assessment and the patient interview, as well as more information on methodological aspects of this study have been published elsewhere [20, 21]. Briefly, the GPs filled in a brief form for each patient, collecting information on general health status and alcohol consumption, AD, as well as any known AD treatment, which the patient might receive.

The patient interview was mainly compiled of several standardized instruments, namely the Composite International Diagnostic Interview (CIDI) to assess DSM-IV AD; the Kessler Psychological Distress Scale (K10) to measure degree of current generic mental distress [22, 23]; the World Health Organization Disability Assessment Schedule 2.0 (WHODAS 2.0) to measure disability [24, 25]; and a questionnaire employed in the UK alcohol treatment trials to collect data on health services utilization [26]. In addition, open questions on alcohol treatment use and smoking were included in the questionnaire.

Both main outcome variables were derived from a combination of the respective questions to the GP and to the patient. As previously shown, both GPs and CIDI had difficulties in recognizing certain AD cases [21]. Therefore, we combined both data sources in order to determine 12-month AD prevalence rates. For the treatment access variable, we gathered information from GP assessment and patient interview as well. While GPs only assessed psychosocial and/or pharmacological AD interventions (exclusively accounting for 41.1 % of all treatment classifications), a more detailed assessment of professional help was included in the patient interview (exclusively accounting for 36.0 % of all treatment classifications). Patients were identified as treatment seekers if they reported having received counselling, pharmacotherapy, individual or group therapy from health professionals, namely GPs, psychotherapists, psychiatrists and other specialists for alcohol problems (e.g. hepatologist, gastroenterologist, neurologist) or medical staff (e.g., in the emergency department) in various settings (e.g., inpatient, outpatient, primary care practices). Overall, we used a wide definition including group therapies led by health professionals, but excluded professionals such as herbalists and priests.

### Statistical methods

Sample size of patients to be assessed by the GPs and to be interviewed were determined a priori: we aimed at minimally 2000 primary care patients being assessed by their GPs in each country. This figure was based on expected AD prevalence rates in primary care settings and their recognition by GPs. A given minimal prevalence of 2.5 % AD cases recognized by GPs would have resulted in about 50 AD cases in each country, adding up to at least 300 AD across all countries – a sufficient sample size to detect small to medium effects with 80 % power [27]. Because all AD cases recognized by the GPs in addition to a random subsample of perceived low risk drinkers and abstainers were supposed to be interviewed, an additional number of AD cases was expected to be identified through the CIDI, resulting in an even greater total sample of AD cases.

In addition to descriptive statistics (all Tables and Web Appendices), different types of regression analyses were used to compare the impact of various influencing variables on different outcomes. Further, t-tests were run to compare group means of independent groups, namely male vs. female (Table 1 & Additional file 1: Web Appendix 1) and cases without treatment vs. cases with treatment (Table 3). Logistic regression was carried out to predict receiving treatment among all AD cases including socio-demographic and health measures as potential predictors (age, sex, below socio-economic average, unemployment, current smoking, Body-Mass-Index, hypertension, liver problems, depression, anxiety, K10 sum score, WHODAS 2.0 sum score, daily amount of alcohol used). All statistical analyses were done taking sampling design into consideration (for details see [20]). To adjust for multiple testing, Bonferroni corrections were used where appropiate. The analyses were conducted using Stata 12.0 [28].

**Table 1:** 12-month prevalence of alcohol dependence diagnoses by sex

	AD diagnosis by GP			AD diagnosis by CIDI^a^			AD diagnosis by GP or CIDI^a^		
	Male	Female	Total	Male	Female	Total	Male	Female	Total
	(N = 5,461)	(N = 7,542)	(N = 13,003)	(N = 3,715)	(N = 5,383)	(N = 9,098)	(N = 3,449)	(N = 5,027)	(N = 8,476)
Percentage diagnosed *% (CI)*	8.7 (8.0 - 9.4)	2.5^c^ (2.2 - 2.9)	5.1 (4.7 - 5.5)	9.4 (8.4 - 10.3)	3.0^c^ (2.5 - 3.4)	5.5 (5.1 - 6.0)	14.6 (13.4 - 15.7)	4.8^c^ (4.2 - 5.3)	8.7 (8.1 - 9.3)
Sought and received professional help^b^ *% (CI)*	28.6 (24.2 - 33.0)	19.6^d^ (13.5 - 25.8)	26.0 (22.4 - 29.7)	18.5 (14.5 - 22.5)	14.8 (9.1 - 20.4)	17.3 (14.0 - 20.6)	24.1 (20.4 - 27.8)	18.6 (13.7 - 23.5)	22.3 (19.4 - 25.2)

*Note.* AD = alcohol dependence. GP = general practitioner. CIDI = Composite International Diagnostic Interview. CI = 95 % confidence interval based on standard error

^a^Data was weighted with inverse sampling probabilities

^b^Percentage of diagnosed patients that sought and received professional help. Data on help seeking behaviour derived from GP assessment in the first three columns, from interview in column four to six and a combined measure from both GP assessment and interview was used in the last three columns

^c^χ^2^-test on sex and diagnosis, all p < .001

^d^χ^2^-test on sex and treatment reception among diagnosed AD cases, p < .05

### Ethical approval

Ethical approvals to carry out the study in all study sites have been obtained from the respective Research Ethic Boards.

- Germany: “Ethikkommission an der TU Dresden“ (Ethics committee at Dresden University of Technology)
- Hungary: “Budapesti Corvinus Egyetem Társadalomtudományi Kara Etikai Bizottság” (Corvinus University of Budapest, Faculty of Social Ethics Committee)
- Italy1 (Friuli-Venezia Giulia): “Comitato Etico Indipendente dell'Azienda per i Servizi Sanitari 2 ‘Isontina’ ” (Independent Ethics Committee of the Company for Health Services n°2 ‘Isontina’)
- Italy2 (Tuscany): “Comitato Etico dell'Azienda Sanitaria Firenze” (Ethical Board of Florence Health Agency)
- Latvia: “Ētikas komitejas Rīgas Austrumu klīniskās universitātes slimnīcas Atbalsta fonds“ (Ethics Committee of the Riga Eastern Clinical University Hospital Support Fund)
- Poland: “Komisja Bioetyczna przy Warszawskim Uniwersytecie Medycznym” (Bioethics Committee at the Medical University of Warsaw)
- Spain: ”Comité Ético de Investigación Clínica. Hospital Clínic de Barcelona” (Hospital Clinic of Barcelona. Ethics Committee for Clinical Research)

## Results

### Participants and descriptive data

Overall, 358 GPs participated, while 478 GPs refused to take part in this study (refusal rate of 56.4 %). The GPs assessed 13,003 patients (5,461 male and 7,542 female, on average: 15.5 patients per day), of which 8,476 patients (3,449 male and 5,027 female) were interviewed. Of all contacted patients, 17.8 % refused to be interviewed. Sample characteristics are published elsewhere [21]. Country variations can be found in Web Additional file 2: Appendix 2.

### Prevalence of alcohol dependence

**Fig. 1:**
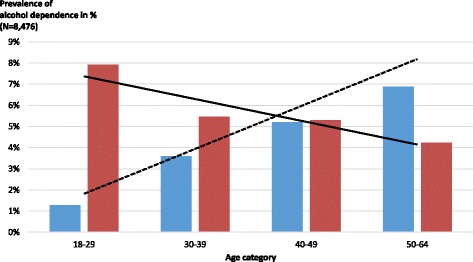
Prevalence of alcohol dependence diagnoses by GP or CIDI, stratified by age categories. Figure displaying age effect on alcohol dependence categories. Legend: (*Blue bars*) Diagnosis by GP, (*Red bars*) Diagnosis by CIDI, (*Dashed line*) Regression line GP diagnosis, (*Continuous line*) Regression line CIDI diagnosis

Table 1 reports 12-month AD prevalence of different ways of identification by sex and includes the respective proportions of patients that sought and received treatment. The AD prevalence as determined by the GP (5.1 %, 95 % confidence interval (CI): 4.7-5.5 %, n = 13,003) was comparable to the prevalence determined by the CIDI (5.5 %, 95 % CI: 5.1-6.0 %, n = 9,098), but the overlap between both was small (18.1 % (95 % CI: 15.6-21.0 %) of all AD cases had both diagnoses). The biggest difference associated with GP vs. CIDI diagnoses was age (see Fig. 1; see [21] for additional differences).

Combining both GP and CIDI derived diagnoses, 8.7 % were identified as alcohol dependent (95 % CI: 8.1-9.3 %, n = 8,476). In all diagnostic categories, the proportion of males was higher than the proportion of females. Considerable regional variation in AD diagnoses could be observed (see Additional file 1: Web Appendix 1). In Italy2 (Tuscany), the lowest rate of AD cases was consistently identified across different diagnostic approaches (GP: 1.8 %, CIDI: 1.5 %, combined: 3.7 %). The largest proportion of diagnoses varied by approach: most GP diagnoses were given in Latvia (7.7 %, 95 % CI: 6.7-8.8 %); CIDI identified most AD cases in Spain (7.6 %, 95 % CI: 6.4-8.7 %); the combined approach resulted in the highest prevalence in Italy1 (11.6 %, 95 % CI: 9.0-14.2 %).

### Comparison of patients with and without alcohol dependence

Cases with a 12-month AD diagnosis, whether identified by the GP or the CIDI, differed markedly from subjects without such a diagnosis on several socio-demographic, behavioural, and health variables (Table 2). Adjusted by sex and age, they were more marginalized (odds ratio (OR) for lower socioeconomic status (SES): 2.07, 95 % CI: 1.76-2.44; for unemployment: 2.63, 95 % CI: 2.20-3.15); more likely to be a smoker (OR: 3.16, 95 % CI: 2.70-3.69); had a higher likelihood to be co-morbid both with physical (OR for liver problems: 7.45, 95 % CI: 5.60-9.90) and mental disorders (OR for depression 2.46, 95 % CI: 1.95-3.09; OR for anxiety: 2.62, 95 % CI: 2.14-3.22), and they had higher scores on the K10 scale for severe mental distress (OR for reaching cut-off: 2.81, 95 % CI: 2.19-3.60). The average number of days in the last 30 days when they were unable to perform work and/or usual daily activities was 2.6 days among people with AD (95 % CI: 2.1-3.0), compared to the non-dependent population with 1.3 days (95 % CI: 1.2-1.5). As expected, people with AD in the past 12 months also had considerable higher average as well as peak alcohol consumption, even though some of them were abstinent at the time of interview (see Table 2).

**Table 2:** Comparison of patients without and with 12-month alcohol dependence on socio-demographic and other variables

	No AD diagnosis (N = 7,656)	AD diagnosis by GP or CIDI (N = 820)	Odds ratio^a^(95 % confidence interval)	Regression coefficient^a^(95 % confidence interval)
Age *mean (SD)*	44.3 (13.1)	45.1 (13.8)		0.79 (−0.20 - 1.78)
SES – self classified *% (CI)* below average	20.1 (19.2 - 21.0)	33.7 (30.4 - 37.1)	2.07 (1.76 - 2.44)^b^	
Unemployed for health or other reason *% (CI)*	11.8 (11.1 - 12.6)	25.2 (22.2 - 28.2)	2.63 (2.20 - 3.15)^b^	
Smoking *% (CI)*	29.5 (28.5 - 30.5)	58.8 (55.4 - 62.3)	3.16 (2.70 - 3.69)^b^	
BMI *mean (SD)*	26.3 (5.2)	25.9 (5.5)		−0.73 (−1.12 - -0.34)^b^
Hypertension *% (CI)*	25.7 (24.7 - 26.7)	33.1 (29.8 - 36.4)	1.30 (1.09 - 1.56)	
Liver problems *% (CI)*	1.9 (1.5 - 2.2)	13.7 (11.3 - 16.1)	7.45 (5.60 - 9.90)^b^	
Depression *% (CI)*	6.9 (6.4 - 7.5)	14.2 (11.7 - 16.6)	2.46 (1.95 - 3.09)^b^	
Anxiety *% (CI)*	9.1 (8.5 - 9.8)	18.7 (15.9 - 21.4)	2.62 (2.14 - 3.22)^b^	
K10				
Above cut-off for serious mental distress % *(CI)*	5.1 (4.6 - 5.6)	12.1 (9.8 - 14.4)	2.81 (2.19 - 3.60)^b^	
Total score *mean (SD)*	6.8 (7.0)	10.5 (8.5)		4.14 (3.55 - 4.74)^b^
WHODAS 2.0				
Number of days of inability to carry out usual activities or work due to health condition *mean (SD)*	1.3 (4.6)	2.6 (6.7)		1.21 (0.74 - 1.68)^b^
Total score *mean (SD)*	8.6 (12.6)	13.7 (16.1)		5.59 (4.47 - 6.70)^b^
Amount of ethanol used daily (in gram)^c^ *mean (SD)*	28.5 (24.7)	57.6 (57.3)		28.44 (23.23 - 33.65)^b^
Chronic heavy drinking^c^ % *(CI)* at least 100 g ethanol daily	2.2 (1.4 - 3.0)	15.8 (12.4 - 19.2)	7.95 (5.10 - 12.40)^b^	
Binge drinking^c^ % *(CI)* at least 200 g ethanol at least weekly	3.4 (2.4 - 4.3)	15.9 (12.5 - 19.3)	5.34 (3.60 - 7.93)^b^	

*Note.* Data was weighted with inverse sampling probabilities

AD = 12-month alcohol dependence, determined by GP & CIDI. GP = general practitioner. CIDI = Composite International Diagnostic Interview. SD = standard deviation. SES = socioeconomic status. CI = 95 % confidence interval based on standard error. BMI = Body-Mass-Index. K10 = Kessler Psychological Distress Scale; cut-off for severe mental distress was 21 points in a total score range from 0 – 40. WHODAS 2.0 = World Health Organization Disability Assessment Schedule 2.0 – total score range: 0 – 100

^a^Regressions are adjusted by sex and age

^b^p significant for Bonferroni-adjusted thresholds (p < .05/16 = 0.003125)

^c^excluding past-year abstainers and low-level drinkers (i.e. drunk less than 10 g pure ethanol per day) from all analyses

### Help seeking

About one in four patients with a current AD diagnosis by GP (26.0 %, 95 % CI: 22.4-29.7 %) and 17.3 % of the patients diagnosed by CIDI (95 % CI: 14.0-20.6 %) sought and received professional help (the proportion for cases defined by either GP or CIDI was 22.3 %, 95 % CI: 19.4-25.2 %). There was considerable country variation in the prevalence of receiving treatment, ranging between 16.6 % (95 % CI: 11.0-22.3 %) in Latvia and 38.5 % in Italy1 (95 % CI: 26.7-50.2 %) for patients diagnosed with AD by GP or CIDI (see Additional file 1: Web Appendix 1). Of all patients receiving professional help, 59.0 % (95 % CI: 52.5-65.5 %) received some kind of treatment in the GP practice.

Compared to male AD patients not seeking treatment, male patients receiving professional help were older (49.0 vs. 44.5 years of age), had more liver problems (28.1 % vs. 10.9 %), were diagnosed more often with anxiety disorders (28.0 % vs. 14.1 %), were more likely to be over the threshold for severe mental distress (20.6 % vs. 8.2 %), had higher K10 (13.4 vs. 8.9 points on a scale ranging 0–40) and WHODAS 2.0 sum scores (18.5 vs. 12.1 points on a scale ranging 0–100), drank more pure alcohol on a daily basis if they did not abstain (90.3 vs. 50.8 gram per day), and had higher proportion of chronic heavy consumption (29.9 % vs. 12.6 % with at least 100 g daily alcohol intake). Comparing female AD patients on the same measures did not yield any significant differences, but all the comparisons were in the same direction (Table 3).

**Table 3:** Socio-demographic and health measures of patients with 12-month alcohol dependence by treatment and sex

	Male patients with AD (N = 558)		Female patients with AD (N = 262)	
	No treatment received (N = 423)	Treatment received (N = 135)	No treatment received (N = 214)	Treatment received (N = 48)
Age *mean (SD)*	44.5 (14.2)	49.0 (11.5)^a^	43.4 (14.7)	47.1 (11.3)
SES – self classified *% (CI)* below average	33.2 (28.6 - 37.9)	37.5 (28.9 - 46.1)	30.6 (24.3 - 37.0)	41.6 (27.1 - 56.1)
Unemployed for health or other reason *% (CI)*	23.5 (19.4 - 27.6)	26.4 (18.8 - 34.1)	26.4 (20.5 - 32.3)	31.5 (18.0 - 44.9)
Smoking *% (CI)*	59.0 (54.2 - 63.8)	69.1 (61.0 - 77.2)	53.3 (46.5 - 60.1)	54.4 (39.8 - 68.9)
BMI *mean (SD)*	26.3 (5.3)	26.6 (6.0)	24.8 (5.5)	25.9 (6.0)
Hypertension *% (CI)*	31.2 (26.7 - 35.7)	44.7 (36.0 - 53.4)	28.0 (21.9 - 34.1)	40.1 (25.6 - 54.7)
Liver problems *% (CI)*	10.9 (8.0 - 13.9)	28.1 (20.1 - 36.1)^a^	7.6 (3.9 - 11.3)	25.7 (12.8 - 38.5)
Depression *% (CI)*	10.5 (7.5 - 13.5)	20.6 (13.6 - 27.6)	13.1 (8.3 - 17.9)	32.5 (18.4 - 46.6)
Anxiety *% (CI)*	14.1 (10.8 - 17.4)	28.0 (20.0 - 35.9)^a^	18.3 (13.0 - 23.5)	34.4 (20.2 - 48.6)
K10				
Above cut-off for serious mental distress % *(CI)*	8.2 (5.5 - 10.9)	20.6 (13.5 - 27.7)^a^	13.9 (9.1 - 18.6)	14.2 (3.2 - 25.2)
Total score *mean (SD)*	8.9 (7.6)	13.4 (9.3)^a^	11.3 (8.9)	12.7 (9.1)
WHODAS 2.0 *mean (SD)*				
Number of days of inability to carry out usual activities or work due to health condition	2.3 (6.4)	4.4 (9.0)	2.2 (5.7)	2.2 (6.4)
Total score	12.1 (15.4)	18.5 (19.4)^a^	13.0 (14.7)	17.5 (17.5)
Amount of ethanol used daily (in gram)^b^ *mean (SD)*	50.8 (46.2)	90.3 (78.8)^a^	43.3 (48.7)	64.9 (63.4)
Chronic heavy drinking^b^ % *(CI)* at least 100 g ethanol daily	12.6 (8.5 - 16.7)	29.9 (20.2 - 39.6)^a^	9.3 (3.1 - 15.4)	23.1 (5.2 - 41.1)
Binge drinking^b^ % *(CI)* at least 200 g ethanol at least weekly	15.6 (11.1 - 20.2)	21.6 (12.8 - 30.4)	10.2 (3.9 - 16.6)	19.2 (2.3 - 3.6)

*Note.* Data was weighted with inverse sampling probabilities.

AD = 12-month alcohol dependence, determined by GP & CIDI. SD = standard deviation. SES = socioeconomic status. CI = 95 % confidence interval based on standard error. BMI = Body-Mass-Index. K10 = Kessler Psychological Distress Scale; cut-off for severe mental distress was 21 points in a total score range from 0 – 40. WHODAS 2.0 = World Health Organization Disability Assessment Schedule 2.0 – total score range: 0 – 100.

^a^p significant for Bonferroni-adjusted thresholds (p < .05/16 tests on the same sample = .003125) in Wald tests comparing AD cases with and without treatment within sex.

^b^excluding past-year abstainers and low-level drinkers (i.e. drunk less than 10 g pure ethanol per day) from all analyses

The multiple logistic regression to predict receiving treatment among all AD cases identified liver problems (OR: 2.43, 95 % CI: 1.46-4.04), K10 sum score (OR: 1.04, 95 % CI: 1.01-1.07) and daily amount of alcohol used (OR: 1.01, 95 % CI: 1.00-1.01) as significant predictors (all factors with p-value < 0.01).

Out of those patients with AD (CIDI or GP, 12-month) that had not received any treatment, 33.5 % gave at least one reason for not doing so: The majority did not consider their drinking and related consequences as a problem (57.0 %, 95 % CI: 50.2-63.8 %). Other major answering categories (multiple answers possible) were shame and stigma (30.0 %, 95 % CI: 23.7-36.3 %), a number of treatment-related barriers such as affordability or lack of information about treatment availability (23.6 %, 95 % CI: 17.8-29.4 %) and the wish to cope with the problem on one’s own (19.2 %, 95 % CI: 13.8-24.6 %). For more details on reasons for not seeking treatment in this study see [29].

## Discussion

### Major findings

Overall, the results confirm that AD (8.7 %; 95 % CI: 8.1-9.3 %) is prevalent in primary care settings, with a prevalence twice as high as the prevalence in general population studies (3.4 %, no CI but only inter quartile range given in the original publication: 0.7-4.7 %; [30]). The higher prevalence may be due to a number of factors, such as different age composition, selection of people with more acute health problems in primary health care, or using two measures in our primary health care settings vs. using one measure in most general population studies (see also [20]). The latter effect can be quantified: using only the CIDI as measure similarly to general population surveys resulted in a prevalence of 5.5 % (95 % CI: 5.1-6.0 %), still considerably higher than from general population surveys, but also lower than the prevalence derived from multiple methods.

We also found a high degree of variability in prevalence between regions, in the case of the two Italian regions even within the same country. Further, we confirmed that the vast majority of cases did not receive professional treatment but treatment is preferably sought by patients with the more severe dependence, with higher levels of alcohol use and mental as well as physical comorbidity.

### Strengths and weaknesses of the study

Our response rate on the individual level with 82.2 % was higher than in current European surveys. Many of our findings were based on self-report and interviews, and the potential bias, while being found relatively low for the instruments used [23, 25, 31–33], can never be excluded. While being representative for the regions selected, we do not and cannot claim representativeness for larger countries or even Europe. The refusal rate at the GP level, even though being over 50 %, seems acceptable when compared with other studies with register-based random sampling [34, 35]. However, it cannot be excluded that the GPs who refused have a different patient population than the participants of this study. Further, the cross-sectional design does not allow for causal inferences of the data.

The careful assessment of each patient by standardised instruments and by the GP is one of the main strengths of the study. This allowed comparisons, and put into perspective the results of general population studies on alcohol use disorders.

### Strengths and weaknesses in relation to other studies, discussing important differences in results

A major finding is that just one in five patients with AD received any formal treatment. This is in line with previous research [8, 15, 36] and has relevant public health implications, since the consequences of untreated AD with respect to mortality and burden of disease are considerable. Further, our results confirm pre-existing knowledge, suggesting that treated cases show higher levels of alcohol and health problems than their untreated counterparts. In a study by Weisner [37], problem drinkers in the general population differed from those in treatment in a number of socioeconomic, drinking and other variables related to social consequences. Our study adds that health problems are especially more prevalent among male patients receiving treatment compared to untreated male cases. For females, this relation did not become significant, partly because of the smaller sample size. While the most severe cases may find their way into treatment, a larger proportion of drinkers with considerable problems still remain untreated. In this context it should be noted that almost 60 % of the patients diagnosed with AD did not consider their drinking as problematic, which might constitute a major reason for low treatment rates.

## Conclusion

### Possible explanations and implications for clinicians and policymakers

As GPs are key to improve recognition and treatment of AD, more efforts are needed to enhance the GPs’ capacity and knowledge to identify patients in need of and to provide the appropriate interventions. Higher recognition and interventions rates for both less severe and severe AD cases in primary care settings could contribute to reduce individual and societal harm.

Younger people with high drinking levels were less identified by GP as compared to the CIDI. While this finding should be confirmed in further studies, there may also be different implications for interventions. For younger adults, a brief intervention to reduce drinking levels may often be best suited. At this point in the life-course, there is less physical co-morbidity, and brief interventions have been shown to be effective in reducing drinking among hazardous and harmful drinkers, including less severe AD in younger adults ([38, 39]; see also [40, 41]). For older people with AD, given the relatively high physical and mental co-morbidity, the GP will have to decide about formal treatment, either in the GP setting or via referral to specialized care. One of the problems here is that standardized guidelines often recommend all or most treatment to happen in specialized care, leaving to GPs only screening, brief interventions for problem drinkers and referral as options [42–44]. As effective treatment options exist including pharmaco-therapeutic options [45, 46], most AD treatment for less severe cases could in principle be done in primary health care.

One way to implement treatment for AD in primary health care would be to handle alcohol use similar to blood pressure, i.e., to routinely check consumption, to suggest options for reduction, and to intervene if certain thresholds are crossed and behavioural alternatives were not successful [47]. Overall, given the high disability associated with AD [4], and the high mortality compared to other mental disorders [5], combined with the fact that reduction of drinking levels is clearly associated with higher survival and less disability [48, 49], there is a strong argument for reducing the current public health impact of AD by increasing intervention rates, including evidence-based formal treatment. This seems possible in primary health care, as in our study GPs demonstrated their ability to detect cases in need for treatment.

### Unanswered questions and future research

With respect to improving the treatment system by shifting AD treatment into primary health care, we need to better specify barriers for intervention in the current systems. The results of research studies up to date seem to be inconsistent, e.g., compare the Swedish results from Stockholm [50] with the results above. This may not be surprising as there is considerable variation across various health care systems in their approach to treat AD across Europe, let alone between North America and Europe [51, 52]. What is needed is a systematic typology for treatment systems and their specific barriers for AD treatment (see also [29]). This promises to be an important step in increasing treatment rates and thus reducing the burden of AD in Europe [1].

## Additional files

Additional file 1: Web Appendix 1. 12-month prevalence of alcohol dependence and treatment seeking behaviour by study site and sex. Table reporting prevalence of alcohol dependence and treatment rates by study site and sex. (DOCX 19 kb) Additional file 2: Web Appendix 2. Socio-demographic and other variables by study site. Table reporting key socio-demographic and health variables by study site. (DOCX 17 kb)

## Abbreviations

AD: alcohol dependence
DSM-IV: Diagnostic and Statistical Manual of Mental Disorders 4th Edition
GPs: general practitioners
EU: European Union
Italy1: Friuli-Venezia Giulia region
Italy2: Tuscany region
CI: confidence interval
CIDI: Composite International Diagnostic Interview
K10: Kessler Psychological Distress Scale
WHODAS 2.0: World Health Organization Disability Assessment Schedule 2.0
UK: United Kingdom
