# Supplementary material for: Alcohol dependence and treatment utilization in Europe – a representative cross-sectional study in primary care
Source: BMC Fam Pract. 2015 Jul 29;16:90. doi: 10.1186/s12875-015-0308-8 (PMC4518612; doi:10.1186/s12875-015-0308-8)
Supplement: Additional file 1: Web Appendix 1. — 12-month prevalence of alcohol dependence and treatment seeking behaviour by study site and sex. Table reporting prevalence of alcohol dependence and treatment rates by study site and sex. (DOCX 19 kb) [file 12875_2015_308_MOESM1_ESM.docx]

| Web Appendix 1  12-months prevalence of alcohol dependence and treatment seeking behaviour by study site and sex | | | | | | | | | |
| --- | --- | --- | --- | --- | --- | --- | --- | --- | --- |
|  | **AD diagnosis by GP** | | | **AD diagnosis by CIDI** | | | **AD diagnosis by GP or CIDI** | | |
|  | Male | Female | Total | Male | Female | Total | Male | Female | Total |
|  | (N=5,461) | (N=7,542) | (N=13,003) | (N=3,715) | (N=5,383) | (N=9,098) | (N=3,449) | (N=5,027) | (N=8,476) |
| **Germany** % *(CI)* | 10.0  (8.1 - 11.8) | 2.5  (1.7 - 3.4) | 5.8  (4.8 - 6.7) | 10.5  (8.0 - 13.1) | 4.1  (2.7 - 5.5) | 6.9  (5.5 - 8.2) | 17.0  (13.9 - 20.1) | 6.4  (4.6 - 8.1) | 11.0  (9.3 - 12.7) |
| Sought and received professional help ^a^ % *(CI)* | 35.4  (25.9 - 44.8) | 21.2  (7.0 - 35.4) | 31.8  (23.8 - 39.8) | 18.7  (8.8 - 28.7) | 0.0  (0.0 - 0.0) | 12.4  (5.5 - 19.2) | 22.3  (13.9 - 30.8)° | 6.7  (0.0 - 14.0) | 17.2  (10.9 - 23.4) |
| **Hungary** % *(CI)* | 7.5  (5.8 - 9.3) | 2.0  (1.2 - 2.7) | 4.1  (3.3 - 4.9) | 7.2  (5.5 - 8.9) | 0.9  (0.4 - 1.4) | 3.3  (2.6 - 4.1) | 11.3  (9.2 - 13.3) | 2.5  (1.7 - 3.3) | 5.8  (4.9 - 6.8) |
| Sought and received professional help ^a^ % *(CI)* | 50.8  (38.3 - 63.3) | 37.5  (17.7 - 57.3) | 47.1  (36.6 - 57.7) | 15.6  (6.7 - 24.5) | 7.7  (0.0 - 22.2) | 14.3  (6.5 - 22.1) | 35.0  (25.6 - 44.3) | 28.6  (13.6 - 43.5) | 33.3  (25.4 - 41.3) |
| **Italy 1 – Friuli-Venezia Giulia** % *(CI)* | 11.3  (8.6 -14.0) | 2.4  (1.2 - 3.6) | 6.5  (5.1 - 8.0) | 10.1  (6.8 - 13.5) | 4.0  (1.5 - 6.5) | 7.0  (4.9 - 9.1) | 17.5  (13.3 - 21.8) | 5.8  (2.8 - 8.8) | 11.6  (9.0 - 14.2) |
| Sought and received professional help ^a^ % *(CI)* | 21.7  (11.1 - 32.2) | 20.0  (0.0 - 41.0) | 21.3  (12.0 - 30.7) | 36.1  (19.5 - 52.8) | 52.9  (21.9 - 83.9) | 41.0  (25.5 - 56.5) | 37.9  (25.1 - 50.7) | 40.1  (13.0 - 67.2) | 38.5  (26.7 - 50.2) |
| **Italy 2 – Tuscany** % *(CI)* | 3.5  (1.7 - 5.3) | 0.7  (0.0 - 1.3) | 1.8  (1.0 - 2.6) | 1.7  (0.0 - 3.4) | 1.4  (0.0 - 2.8) | 1.5  (0.5 - 2.6) | 5.6  (2.6 - 8.6) | 2.3  (0.6 - 4.1) | 3.7  (2.1 - 5.3) |
| Sought and received professional help ^a^ % *(CI)* | 37.5  (1.6 - 73.4) | 25.0  (0.0 - 74.1) | 33.3  (5.4 - 61.3) | 22.9  (0.0 - 63.0) | 26.8  (0.0 - 71.3) | 25.0  (0.0 - 55.4) | 27.9  (1.6 - 54.2) | 28.1  (0.0 - 61.6) | 28.0  (7.3 - 48.7) |
| **Latvia** % *(CI)* | 12.7  (10.6 - 14.9) | 4.7  (3.6 - 5.7) | 7.7  (6.7 - 8.8) | 10.0  (7.4 - 12.6) | 2.8  (1.7 - 3.9) | 5.3  (4.1 - 6.5) | 19.0  (15.6 - 22.4) | 6.4  (4.8 - 7.9) | 10.8  (9.2 - 12.4) |
| Sought and received professional help ^a^ % *(CI)* | 9.4  (4.1 - 14.7) | 5.8  (0.2 - 11.4) | 8.1  (4.1 - 12.9) | 19.3  (8.4 - 30.2) | 10.3  (0.0 - 21.6) | 16.2  (8.0 - 24.4) | 17.6  (10.2 - 25.0) | 15.1  (6.4 - 23.9) | 16.6  (11.0 - 22.3) |
| **Poland** % *(CI)* | 7.7  (6.2 - 9.3) | 2.3  (1.5 - 3.1) | 4.9  (4.0 - 5.7) | 7.8  (5.4 - 10.2) | 4.8  (3.2 - 6.4) | 6.0  (4.7 - 7.3) | 15.5  (12.6 - 18.5) | 6.5  (4.8 - 8.2) | 10.1  (8.5 - 11.7) |
| Sought and received professional help ^a^ % *(CI)* | 40.5  (24.5 - 56.6) | 71.4  (35.2 - 100.0) | 45.5  (30.5 - 60.4) | 14.4  (3.5 - 25.3) | 23.5  (9.2 - 37.7) | 18.8  (9.8 - 27.8) | 18.4  (10.7 - 26.1) | 21.5  (10.1 - 32.9) | 19.6  (13.1 - 26.1) |
| **Spain** % *(CI)* | 4.7  (2.9 - 6.4) | 1.3  (0.6 - 2.1) | 2.7  (1.8 - 3.6) | 12.9  (10.6 - 15.2) | 3.8  (2.7 - 4.9) | 7.6  (6.4 - 8.7) | 14.6  (11.7 - 17.5) | 4.5  (3.1 - 6.0) | 8.6  (7.1 - 10.1) |
| Sought and received professional help ^a^ % *(CI)* | 32.0  (13.3 - 50.7) | 27.3  (0.0 - 54.9) | 30.6  (15.3 - 45.8) | 17.0  (9.8 - 24.1) | 13.3  (3.4 - 23.3) | 15.9  (10.1 - 21.7) | 17.3  (9.1 - 25.5) | 16.2  (4.3 - 28.1) | 17.0  (10.2 - 23.7) |
| *Note*. AD = alcohol dependence. GP = general practitioner. CIDI = Composite International Diagnostic Interview. CI = 95% confidence interval based on standard error.  Χ² tests on sex and treatment seeking among AD cases did not yield any significant results (Bonferroni-adjusted: α=0.05 / 7 tests = 0.0071)  ^a^ Percentage of diagnosed patients that sought and received professional help. Data on help seeking behaviour derived from GP assessment in the first three columns, from interview in column three to six and a combined measure from both GP assessment and interview was used in the last three columns. | | | | | | | | | |
