# Supplementary material for: Alcohol dependence and treatment utilization in Europe – a representative cross-sectional study in primary care
Source: BMC Fam Pract. 2015 Jul 29;16:90. doi: 10.1186/s12875-015-0308-8 (PMC4518612; doi:10.1186/s12875-015-0308-8)
Supplement: Additional file 2: Web Appendix 2. — Socio-demographic and other variables by study site. Table reporting key socio-demographic and health variables by study site. (DOCX 17 kb) [file 12875_2015_308_MOESM2_ESM.docx]

| **Web Appendix 2**  **Socio-demographic and other variables by study site** | | | | | | | |
| --- | --- | --- | --- | --- | --- | --- | --- |
|  | **Germany ^a^**  (N=2,304  GP assessments; N=1,356 interviews) | **Hungary ^a^**  (N=2,308  GP assessments;  N=2,306 interviews) | **Italy 1 – Friuli-Venzia Giulia ^a^**  (N=1,149  GP assessments;  N=492 interviews) | **Italy 2 – Tuscany ^a^**  (N=1,005  GP assessments;  N=451  interviews) | **Latvia ^a^**  (N=2,468  GP assessments;  N=1,302  interviews) | **Poland ^a^**  (N=2,396  GP assessments; N=1,197 interviews) | **Spain ^a^**  (N=1,373  GP assessments;  N=1,994  interviews) |
| **Age** (GP ^b^) *mean (SD)* | 45.0 (13.4) | 42.9 (13.7) | 47.6 (12.2) | 47.3 (11.8) | 44.5 (12.9) | 43.9 (13.3) | 43.2 (12.9) |
| **Sex** (GP ^b^) *% (CI)* |  |  |  |  |  |  |  |
| Female | 56.6 (54.5 - 58.6) | 61.5 (59.5 - 63.5) | 53.7 (50.8 - 56.6) | 59.9 (56.9 - 62.9) | 61.8 (59.9 - 63.7) | 52.5 (50.5 - 54.5) | 58.8 (56.7 - 61.0) |
| **Married or cohabiting**  (self ^b^) *%* *(CI)* | 70.8 (68.4 - 73.3) | 58.8 (56.8 - 60.8) | 65.0 (60.5 - 69.6) | 75.1 (71.1 - 79.1) | 58.7 (56.0 - 61.4) | 71.7 (69.1 - 74.3) | 63.3 (61.2 - 65.5) |
| **Employed** (self ^b^) *%* *(CI)*  Paid work or self-employed | 57.2 (54.6 - 59.9) | 59.2 (57.2 - 61.2) | 62.0 (57.4 - 66.7) | 62.2 (57.7 - 66.6) | 64.8 (62.2 - 67.5) | 71.3 (68.7 - 73.9) | 61.9 (59.8 - 64.0) |
| **Unemployed** (self ^b^) *%* *(CI)*  Health or other reason | 12.4 (10.6 - 14.1) | 10.1 (8.9 - 11.4) | 2.0 (0.6 - 3.5) | 1.5 (0.4 - 2.7) | 20.2 (18.0 - 22.4) | 11.7 (9.8 - 13.5) | 19.6 (17.9 - 21.4) |
| **SES – self classified** *%* *(CI)* |  |  |  |  |  |  |  |
| Above average | 20.1 (18.0 - 22.3) | 2.1 (1.5 - 2.7) | 8.8 (6.0 - 11.5) | 2.9 (1.4 - 4.5) | 6.0 (4.7 - 7.3) | 5.3 (4.0 - 6.7) | 6.9 (5.8 - 8.0) |
| Average | 69.0 (66.5 - 71.5) | 75.8 (74.1 - 77.6) | 74.5 (70.3 - 78.7) | 76.0 (72.0 - 79.9) | 67.1 (64.5 - 69.7) | 83.4 (81.2 - 85.6) | 58.0 (55.8 - 60.1) |
| Below average | 10.9 (9.2 - 12.5) | 22.1 (20.4 - 23.8) | 16.7 (13.1 - 20.3) | 21.1 (17.3 - 24.9) | 26.9 (24.4 - 29.3) | 11.3 (9.4 - 13.1) | 35.1 (33.0 - 37.2) |
| **Education** in years (self ^b^)  *mean (SD)* | 13.5 (3.0) | 12.3 (3.0) | 11.6 (3.6) | 11.9 (4.2) | 13.1 (3.1) | 13.4 (3.0) | 12.4 (4.7) |
| **BMI** (GP ^b^) *mean (SD)* | 26.3 (5.2) | 26.0 (4.9) | 25.5 (4.7) | 24.7 (4.3) | 26.8 (5.7) | 26.5 (5.1) | 26.1 (5.4) |
| **Hypertension** (GP ^b^)  *%* *(CI)* | 28.5 (26.7 - 30.4) | 27.3 (25.4 - 29.1) | 25.7 (23.1 - 28.2) | 17.0 (14.7 - 19.3) | 29.1 (27.3 - 30.8) | 37.2 (35.1 - 39.3) | 15.3 (13.4 - 17.2) |
| **WHODAS 2.0** at least one day of reduced activities (self ^b^) *%* *(CI)* | 40.9 (38.3 - 43.6) | 20.2 (18.5 - 21.8) | 26.7 (22.5 - 30.8) | 21.0 (17.3 - 24.8) | 35.6 (33.0 - 38.3) | 21.2 (18.8 - 23.5) | 44.5 (42.3 - 46.7) |
| **Severe distress** *%* *(CI)* |  |  |  |  |  |  |  |
| Depression or anxiety (GP ^b^) | 12.5 (11.2 - 13.9) | 7.9 (6.8 - 9.0) | 21.8 (19.5 - 24.2) | 15.4 (13.2 - 17.7) | 17.1 (15.6 - 18.6) | 12.3 (10.9 - 13.7) | 22.8 (20.6 - 25.1) |
| Mental distress assessed by K10 (self ^b^) | 8.2 (6.7 - 9.6) | 5.3 (4.4 - 6.2) | 4.5 (2.7 - 6.2) | 3.1 (1.5 - 4.7) | 3.2 (2.2 - 4.2) | 5.1 (3.9 - 6.4) | 8.3 (7.1 - 9.5) |
| **Alcohol use last** **12 months**  (self ^b^) *%* *(CI)* | 84.7 (82.7 - 86.7) | 62.0 (60.0 - 63.9) | 63.0 (58.2 - 67.9) | 66.2 (61.8 - 70.6) | 56.3 (53.6 - 59.1) | 58.5 (55.6 - 61.3) | 62.3 (60.1 - 64.4) |
| **Currently smoking**  (self ^b^) *%* *(CI)* | 32.4 (29.9 - 34.9) | 36.0 (34.1 - 38.0) | 27.8 (23.7 - 32.0) | 21.4 (17.6 - 25.2) | 34.0 (31.4 - 36.6) | 28.9 (26.3 - 31.5) | 29.7 (27.7 - 31.7) |
| *Note.* GP = general practitioner. SD = standard deviation. CI = 95% confidence interval based on standard error. SES = socioeconomic status. BMI = Body-Mass-Index. WHODAS 2.0 = World Health Organization Disability Assessment Schedule 2. K10 = Kessler Psychological Distress Scale; cut-off for severe mental distress was 21 points in a total score range from 0-40.  ^a^ Different reference groups apply, based on the source of the variable: For variables stemming from physician assessment, all patients that were assessed by the general practitioner were analysed unweighted. For interview-based measures, all interviewed patients were analysed and weighted with inverse sampling weights.  ^b^ ‘self’ indicates that data originates in interview while ‘GP’ indicates that data stems from general practitioner assessment. | | | | | | | |
